# Vitamin D: Current Challenges between the Laboratory and Clinical Practice

**DOI:** 10.3390/nu13061758

**Published:** 2021-05-21

**Authors:** Ludmila Máčová, Marie Bičíková

**Affiliations:** Institute of Endocrinology, Národni 8, 11694 Prague, Czech Republic; mbicikova@endo.cz

**Keywords:** vitamin D, metabolites, VDR, determination, LC–MS/MS, genomic effects, non-genomic effects

## Abstract

Vitamin D is a micronutrient with pleiotropic effects in humans. Due to sedentary lifestyles and increasing time spent indoors, a growing body of research is revealing that vitamin D deficiency is a global problem. Despite the routine measurement of vitamin D in clinical laboratories and many years of efforts, methods of vitamin D analysis have yet to be standardized and are burdened with significant difficulties. This review summarizes several key analytical and clinical challenges that accompany the current methods for measuring vitamin D. According to an external quality assessment, methods and laboratories still produce a high degree of variability. Structurally similar metabolites are a source of significant interference. Furthermore, there is still no consensus on the normal values of vitamin D in a healthy population. These and other problems discussed herein can be a source of inconsistency in the results of research studies.

## 1. Introduction

Vitamin D is one of the most frequently discussed nutrients in contemporary public and scientific medicine. Thousands of new scientific papers every year demonstrate that the subject is constantly evolving, and the current severe acute respiratory syndrome coronavirus 2 (SARS-CoV-2) pandemic has further increased interest because vitamin D deficiency has been reported as a risk factor for worse courses of coronavirus disease. Experts from around the world are in agreement that vitamin D deficiency is a global health problem, even in areas with adequate year-round sun exposure. This unfavorable situation is further exacerbated by widespread concerns about the harmfulness of sunlight, the use of sunscreens, and the sedentary lifestyle of current societies with a tendency to spend more time indoors.

Many questions regarding vitamin D have not yet been satisfactorily answered. In addition to calcium/phosphate balance, vitamin D also regulates many other systems in the human body such as the immune, endocrine, cardiovascular, and nervous systems. The vitamin D receptor (VDR) and the enzymes necessary for vitamin D synthesis (specific hydroxylases, epimerases, etc.) have been observed in more than 35 types of cells throughout the human body [1,2], which indicates the pleiotropic effect of vitamin D. Hence, in addition to its well-described function in maintaining calcium/phosphate metabolism, which has been known since 1920s, studies performed over the past 20 years have demonstrated the beneficial role of vitamin D in numerous widespread diseases, including metabolic [3], cardiovascular [4], immune [4], and neuropsychiatric diseases [5,6], as well as cancer [4], the current coronavirus disease [7,8], and other conditions [9]. Though most studies have highlighted the beneficial effects of vitamin D, some studies on this compound have not observed any effects.

With the expanding number of published studies, awareness of vitamin D is increasing and the need for laboratory testing is rising. Due to vitamin D’s lipophilic nature, tendency to bind to a protein transporter, and extremely low concentrations, its measurement is accompanied by several analytical complications. Here, we discuss several challenges that accompany current clinical and laboratory testing of vitamin D.

## 2. Vitamin D Metabolites and Their Clinical Significance

### 2.1. Major Vitamin D Metabolites

Vitamin D metabolism involves a complex network of metabolic processes with more than 50 structurally similar metabolites [10]. In brief, the major metabolic pathways are based on two sources of vitamin D: cholecalciferol (vitamin D3) is produced in the cutaneous tissue of animals, and ergocalciferol (vitamin D2) is synthesized in plants. Both forms first undergo hydroxylation at position 25 to create calcidiol (25(OH)D), which predominantly occurs in the liver. This step is performed by several enzymes from the cytochrome P450 family with 25-hydroxylase activity. To date, at least six enzymes (sterol 27-hydroxylase—CYP27A1, cytochrome P450 3A4—CYP3A4, vitamin D 25-hydroxylase—CYP2R1, cytochrome P450 2C11—CYP2C11, cytochrome P450 2J1—CYP2J1, and vitamin D3 25-hydroxylase—CYP2D25) that possess 25-hydroxylase activity have been identified, as reviewed by Jenkinson [11]. The need for multiple back-up enzymes indicates that the 25-hydroxylation of vitamin D is absolutely crucial for the normal functioning of the human body.

The second hydroxylation occurs via the action of vitamin D 1α-hydroxylase (CYP27B1) at position 1 to produce calcitriol (1α,25(OH)2D), which predominantly occurs in the kidney. However, other tissues, including the placenta [12], immune cells, enterocytes, prostate cells, and pancreatic cells [13], are known to express CYP27B1 and thereby be involved in the local production of calcitriol. Calcitriol is the only form of vitamin D that is commonly recognized as biologically active, although it is very likely that other metabolites also have calcemic or non-calcemic effects [14]. However, plasma calcitriol only reaches picogram/milliliter concentrations, and its biological half-life is only calculated in hours, which reflects the activity of 1α-hydroxylase in the kidney.

Currently, the measurement of total 25(OH)D is considered to be more clinically relevant for monitoring vitamin D supply in patients. Calcidiol is a major derivative of vitamin D, occurs in plasma at concentrations that are thousands of times higher than those of calcitriol, has a biological half-life of dozens of days, and generally better reflects vitamin D saturation in an organism.

### 2.2. Vitamin D Epimers

All major metabolites of vitamin D can be irreversibly converted by an epimerase at their C3 position in a reaction that occurs in the liver, bone, or skin cells [15]. The exact source and biological activity of the epimers have not yet been identified, but a higher proportion of C3-epimers (up to 61.1% of the total vitamin D) has been detected in mothers and newborns [16,17]. These observations indicate the importance of epimers in pregnancy and early development. The weak correlation between maternal and neonatal 3-epi-25(OH)D3 suggests that C3-epimers have an endogenous fetal origin rather than a maternal one [18].

C3-epimers of vitamin D also have plausible roles in inflammatory diseases, as significantly lower concentrations of these alternative serum metabolites have been observed in patients with rheumatoid and reactive arthritis [19]. Other studies have revealed the calcemic regulatory effect of 3-epi-1α,25(OH)2D3, but this effect has been less pronounced than that of its non-epimeric form [20]. However, in some cases, 3-epimers have displayed equal or even stronger activity relative to their non-epimeric counterparts [10,15].

After the discovery of C3-epimers, an epimer in the C1 position was accidentally revealed during the optimization of a chromatographic method. The co-eluting isobar was identified as 1β,25(OH)2D3 and appeared with a median value of 10.56 pg/mL in the serum of healthy volunteers [21]. The origin of C1-epimers is unclear, but the C1-β-hydroxylation of other compounds is predicted to occur in humans. Similarly, Wang et al. fortuitously identified 4β,25(OH)2D3 as a novel substance that co-eluted with commonly investigated metabolites at concentrations similar to those of 1,25(OH)2D3 [22].

### 2.3. Catabolites of Vitamin D

Vitamin D is inactivated by a multistep pathway catalyzed by vitamin D 24-hydroxylase (CYP24A1). This enzyme has been detected in various target tissues, including the placenta [23], brain [24], kidneys, intestines, and bone [25]. Both 25(OH)D3 and 1,25(OH)2D3 are initially hydroxylated at C24 or C23, followed by C24-oxidation and C23-oxidation pathways that lead to their excretory products, namely calcitroic acid and 1α,25(OH)2D3-26,23-lactone, respectively [26]. Though lactones are primarily catabolic products, and they have biological functions in bone resorption. Interestingly, 24-oxo metabolites were observed to be significantly more potent bone-resorbing agents than lactones, which suggests that conversion to lactones represents a substantial inactivation step, whereas conversion to 24-oxo-derivatives results in less of a reduction in biological activity [27]. The intermediate 24,25(OH)2D3, which occurs in plasma at concentrations on the order of ng/mL, is the most abundant dihydroxy-vitamin D metabolite in the human circulation [28] and appears to have a physiological role in the repair of bone fractures and the development of growth plates without the involvement of the VDR [29].

The activity of CYP24A1 determines the rate of degradation and thus the amount of bioactive vitamin D. CYP24A1 is tightly regulated by 1α,25(OH)2D3, plasma calcium, and parathormone. However, its activity also increases with age and in some non-physiological conditions [4]. It is of interest that an increased activity of CYP24A1 has been observed in different cancers [30,31,32], and *CYP24A1* has been identified as a proto-oncogene [33,34].

### 2.4. Conjugates of Vitamin D

Conjugation is a mechanism that changes the solubility of compounds, which alters their biological activity and the probability of their elimination from the organism. Sulfation is performed by the enzyme sulfotransferase (SULT), and this process was thoroughly described, inter alia, for steroidal dehydroepiandrosterone and its sulfate [35]. Vitamin D and its metabolites are typically converted by the subtype SULT2A1, and the rate of sulfation is associated with the gene variant encoding the enzyme [36]. 25(OH)D3-3-sulfate, with a mean concentration of 16.7 ng/mL, was identified as the most abundant sulfated form of vitamin D in serum, with levels often exceeding those of unconjugated 25(OH)D3 [37]. Other vitamin D sulfates (25(OH)D2-sulfate and vitamins D2- and D3-sulfate) were detected in human serum (on the order of 0.2–0.6 ng/mL) [38] but not in urine, which raised the hypothesis that these sulfate metabolites serve as 25(OH)D3 reservoirs [36] and are secreted via bile.

Glucuronidation is another conjugation process for vitamin D and its metabolites, and it is mediated by UDP-glucuronosyltransferases (UGT). The most abundant glucuronide of vitamin D in the circulation is 25(OH)D3-glucuronide, which reaches average concentrations of 1.36–2.4 ng/mL [39,40]. On the contrary, one of the major urinary vitamin D3 metabolites in humans is 24,25(OH)2D3-glucuronide (detected in plasma at extremely low concentrations of up to 120 ng/mL), which corresponds to 52.8 ± 19.3 ng/g creatinine [41].

### 2.5. Rare and Predicted Vitamin D Metabolites

New metabolites of vitamin D are constantly being revealed with the extensive development of advanced analytical technologies that provide the ability to both characterize the exact stereo-chemical structure and detect concentrations in picograms or even femtograms per milliliter. In some cases, such metabolites have been discovered by chance, such as the previously mentioned epimers 1β,25(OH)2D3 and 4β,25(OH)2D3. The biological functions of these substances are yet to be discovered, although some of their hormonal functions have been demonstrated in animal models or in vitro.

Furthermore, there is a large group of vitamin D metabolites that has not yet been detected in the human circulation; this is strongly predicted to be the case based on the current knowledge of enzymatic and physiological processes. A recently published extensive review by Tuckey et al. provided an illustrative overview [10].

### 2.6. Free Vitamin D

According to the free hormone hypothesis established for steroid and thyroid hormones, only unbound or weakly bound fractions of hormones are considered to be “bioavailable.” Because of the lipophilic character of vitamin D, 85–90% of circulating hormones are tightly bound to a carrier protein (vitamin D binding protein (DBP)), 10–15% are weakly bound to albumin, and less than 0.1% circulates in the unbound form [42,43]. In addition to facilitating circulatory transport, DBP also protects bound vitamin D metabolites from rapid renal excretion. These metabolites are also preferably used by the aforementioned conjugates [36].

The importance of measuring the free fraction of vitamin D has been re-evaluated several times [44,45]. Studies have shown that the measurement of total vitamin D is not sufficient for evaluating the status of this nutrient in patients, and the determination of free vitamin D has been confirmed to be beneficial, at least under certain conditions [46,47].

### 2.7. Vitamin D Supply and Absorption

Vitamin D supplementation is often necessary due to the insufficient synthesis of vitamin D in the skin after exposure to ultraviolet B radiation. Therefore, a growing body of interest in the absorption and metabolism of orally ingested vitamin D supplements has been observed. Until recently, vitamin D was thought to be absorbed by a simple passive diffusion process, but recent studies revealed that these are more likely mechanisms involving membrane carriers. Both ergocalciferol and cholecalciferol are rapidly absorbed after oral intake, with the maximum level detected 24 h after administration. Plasma concentrations of 25(OH)D also increase, with the highest levels achieved after approximately 7–14 days, depending on the dose of vitamin D administered [48]. Moreover, 25(OH)D has been reported to be better absorbed than the non-hydroxy vitamin D forms—cholecalciferol and ergocalciferol [49].

### 2.8. Physiological and Clinical Significance of Vitamin D Metabolites—A Review

Even though over 50 different vitamin D metabolites have been described so far, which enables us to speak of a whole vitamin D metabolome, only 1α,25(OH)2D has been generally recognized as biologically active. By consensus, the determination of total 25(OH)D has been used to evaluate the vitamin D supply. The physiological effects of other metabolites are only considered potential, as their roles in vivo remain unrecognized. C-3 epimers of vitamin D are the exception for which a weak calcemic and immunomodulatory effect has been demonstrated and whose ratio to total circulating vitamin D is a promising tool for predicting disease status such as type 1 diabetes, rheumatoid arthritis, and Alzheimer disease [50]. The 24,25(OH)2D to total 25(OH)D ratio is used as a marker for vitamin D catabolism and as a predictor of response to vitamin D supplementation [51]. Relatively high serum levels of 25(OH)D-3-sulfate and the ability to be converted to unconjugated 25(OH)D suggest its role as a reservoir of unconjugated forms. On the contrary, conjugated glucuronides, which predominate in urine, serve to monitor vitamin D excretion. In addition, the determination of a number of vitamin D metabolites may be useful in identifying possible genetic polymorphisms and variations, especially when the mutation does not cause a disease or an apparent phenotype [10].

## 3. Vitamin D Determination

Though the measurement of vitamin D is predominantly performed on blood samples obtained by venipuncture in clinical practice (for diagnostic/therapeutic purposes), for research purposes, vitamin D is measured in other biological matrices, such as urine [41], tissues [52,53], tissue culture cells, umbilical cord blood [54,55,56], finger-prick blood [57], amniotic fluid [58,59], breastmilk [38], and synovial fluid [19]. Table 1 lists vitamin D metabolites that have been detected in different biological matrices in a wide concentration range from a few picograms to dozens of nanograms per milliliter of liquid sample.

Due to the vitamin’s extremely low circulating concentrations, lipophilic properties, VDBP binding, and other matrix interactions, the measurement of vitamin D in biological samples requires pre-analytical treatment to reduce the adverse effects of the matrix. Different methods and conditions are used for sample preparation prior to vitamin D analysis, but the most common techniques are protein precipitation (PP), liquid–liquid extraction (LLE), solid-phase extraction (SPE), or their combination, as summarized in Table 2.

In the last two decades, the determination of circulating vitamin D has become a standard routine examination in biochemical laboratories. In general, vitamin D measurement methods can be divided into two main approaches: methods based on immunoassays (CLIA, ECLIA, RIA, and ELISA) and chromatographic methods (HPLC and LC–MS).

### 3.1. Immunoassays

Due to the advantages of automation and rapid results, immunoanalytical methods are the most frequently used techniques for vitamin D measurement in clinical laboratories. The disadvantages of these methods are the non-specificity of the used antibodies and significant interference. As a result of these limitations, most of these methods are not able to quantify individual forms of vitamin D. The cross-reactivity between similar metabolites can be a source of inaccuracies that lower the specificity of the method. It follows that the quality of the used antibody defines the quality of the assay. Furthermore, some of these methods designed for vitamin D measurement are based on the use of DBP, which binds a number of vitamin D metabolites with different affinities. A comparison of different methods used in different laboratories showed that assays produced significantly dissimilar results, indicating that the measurement of vitamin D supply is a function of the laboratory [74].

### 3.2. Chromatographic Methods

The general advantage of chromatographic methods is their ability to efficiently separate and quantify structurally similar metabolites. However, these techniques are also burdened with certain limitations and have their own drawbacks, especially the complex technical equipment and the time-consuming preparation and evaluation of samples.

Initially, chromatographic methods for vitamin D measurement combined thin-layer chromatography (TLC) with gas chromatography (GC) [75]. Several years later, high-performance liquid chromatography with ultraviolet detection (HPLC–UV/VIS) was introduced [76]. Currently, with the advanced development of analytical methods, LC–MS/MS is accepted as an alternative method, especially in research laboratories. Though a number of promising approaches for vitamin D measurement have been reported, this challenge remains unresolved. An overview of available mass spectrometry assays, in which the authors compared techniques such as the type of chromatographic column, mobile phases, type of ionization, and use of derivatization, was recently published [77].

Due to the picogram (in mL) amounts of some vitamin D analytes, derivatization techniques are often employed to increase the ionization efficiency and analytical sensitivity of the methods. In general, three types of approaches are currently used to improve the ionization of vitamin D metabolites. A significant group comprises methodologies that use Cookson-type reagents (also known as dienophiles), which include 1,2,4-triazoline-3,5-dione (TAD), 4-phenyl-1,2,4-triazole-3,5-dione (PTAD), substituted TAD (DMEQ-TAD, DAPTAD, Ampliflex Diene, and SecoSET. Several dienophiles improve sensitivity by 10–100-fold and enable detection at the picogram/microliter range in 25 microliter samples. However, an important feature of tagging with a dienophile is that two adduct peaks are formed when the reagent attacks the molecule from either plane of the cis-triene [78]. Another type of method involves 2-nitrosopyridine (PyrNO) and other functionalized nitroso compounds that improve ionization and lead to even higher sensitivity than PTAD [79]. Both previously mentioned derivatization techniques target the s-cis-diene structure of the vitamin D molecule to create Diels–Alder adducts. The last type of approach involves the acylation of the C3-hydroxyl of the vitamin D molecule with isonicotinoyl chloride (INC) [57]. Using this derivatization method, the authors observed no isomer interference and an improvement in the detection sensitivity of 200–1000-fold, which enabled the quantification of the most challenging metabolites at concentrations as low as 1 ng/mL, even in finger-prick blood samples (3 µL of plasma).

### 3.3. Standardization

The Vitamin D External Quality Assessment Scheme (DEQAS) was launched in London in 1989 with the aim of ensuring the analytical reliability of assays. The initial focus was 25(OH)D, followed by the inclusion of 1,25(OH)2D in 1992. A pilot scheme for 24,25(OH)2D was launched in 2015, and the evaluation of results of free 25(OH)D were added in October 2019.

To standardize the laboratory measurement of vitamin D status, the US National Institutes of Health (NIH) Office of Dietary Supplements established the Vitamin D Standardization Program (VDSP) in 2010. Since then, the VDSP, in cooperation with the National Institute of Standards and Technology (NIST), Ghent University Reference Measurement Procedures, and the United States Centers for Disease Control and Prevention (CDC), has developed a reference measurement system to establish the international standardization of 25(OH)D measurement [68,69,80]. In 2015, the NIST developed a reference measurement procedure for the determination of (24R),25(OH)2D3 in human serum using isotope-dilution LC–MS/MS [81].

For the validation of in-house methods, the NIST released Standard Reference Materials^®^ (SRMs) with NIST-certified values. SRMs can be used as “trueness” controls of vitamin D assays, e.g., SRM 972a, or as calibrators (SRM 2972). The VDSP has also developed protocols for calibrating previous measurements of 25(OH)D to the current gold standard reference measurement procedures [82,83]. Due to the considerable difference in results before and after standardization, experts from the VDSP strongly recommend suspending the publication of meta-analyses based on unstandardized 25(OH)D data [84].

Since October 2012, the NIST and VDSP have worked closely with the DEQAS by using the reference measurement procedure to analyze quarterly serum sample sets and assign an accuracy-based target value for total serum 25(OH)D. In 2017, the DEQAS switched to CDC for target value assessment [85].

According to the recent reports of the DEQAS, over 1000 participants from 56 countries were involved in this external quality testing, in which 30 different methods or their variants were used. Despite 10 years of standardization efforts, the measurement of vitamin D still faces problems with cross-reactivity and matrix effects, e.g., the presence of triglycerides [86] or biotin [87]. In 2018, the difference between results from the NIST and tested participants still varied by up to 23.7%. However, the bias of LC–MS/MS methods was much lower than that of antibody-based 25(OH)D methods [87].

The US NIST and the NIH also established the Vitamin D Metabolites Quality Assurance Program (VitDQAP), which was used to perform an inter-laboratory comparison of vitamin D evaluation methods in human serum and plasma and to assess measurements made via in-house methods. This program was concluded in 2015, and since 2017, some of the VitDQAP functions have been served through the NIST Health Assessment Measurements Quality Assurance Program (HAMQAP) [88].

Other systems for the external quality assessment of vitamin D measurement—such as the German Reference Institute for Bioanalytics (RfB) or the Czech equivalent, SEKK—are used in Europe. According to the latest published report (August 2020), luminescence methods are still the most frequently used to determine both total 25(OH)D and 25(OH)D3 alone. The coefficient of variation between laboratories was found to range from 5 to 35% in individual groups, depending on the used analytical method [89].

## 4. Clinical Aspects of Vitamin D

### 4.1. Physiological Ranges

Difficulties in the accurate determination of this analyte are also related to the evaluation of reference values for a healthy population. There is still no consensus on adequate levels of vitamin D. Decades ago, the optimal circulating vitamin D concentration was preliminarily assessed based on the parathyroid hormone response. This approach is no longer accepted by some scientific experts, who recommend re-evaluating the normal ranges with respect to the extra-skeletal effects of vitamin D, e.g., immunomodulation [90] and normoglycemia [91]. Therefore, clinical experts and authorities use different cut-offs to define hypovitaminosis (Table 3). In agreement with the Institute of Medicine guidelines, some studies have used 20 ng/mL as a cut-off for vitamin D insufficiency, but other studies have followed the recommendation of the Endocrine Society, which defines levels below 29 ng/mL as insufficient [84]. Furthermore, the manufacturers of analytical kits reference different normal ranges depending on the used methodology [74,90]. Importantly, variations in the effect thresholds of 25(OH)D may serve as potential confounders when evaluating the outcomes of clinical trials [92].

Despite the efforts of experts and authorities, optimal physiological values have not yet been determined for the most at-risk groups, such as pregnant women, children, and the elderly. For example, some authors have suggested that the optimal 25(OH)D level for pregnant women is higher than 40 ng/mL [93]. In contrast, there is quite a consensus among researchers, as well as data support, that the 25(OH)D concentration must rise above 750 nmol/L (~300 ng/mL) to produce vitamin D toxicity, although an upper limit of 250 nmol/L (~100 ng/mL) is recommended to ensure safety margins [94].

As mentioned earlier, vitamin D has a number of functions across the human body. It reduces the severity of more than 70 different diseases, which opens debate about the optimal serum levels of vitamin D for various aspects of patient’s health. Though optimal levels of 20–32 ng/mL have been proposed for musculoskeletal benefits for immunomodulatory and anticancer properties, vitamin D concentrations above 50 ng/mL have been recommended [9].

As vitamin D is formed in the skin after sun exposure, its synthesis depends on latitude and seasonal variation. Indeed, no vitamin D synthesis in latitudes higher than 50° has been reported in the winter and spring months [95]. Other studies have found the highest hypovitaminosis D during winter and spring when 25(OH)D concentration was suboptimal (<30 ng/mL) in 87.1% of participants compared to 60.9% in summer and fall [96].

### 4.2. Genomic vs. Non-Genomic Effects of Vitamin D

The classical characterization of bioactive vitamin D (1,25(OH)2D3) as a hormone that modulates calcium homeostasis through the nuclear VDR, thus affecting gene expression, has been expanded in recent years. In addition to long-term genomic effects (response in several hours to days) that alter gene transcription (about 3% of the human genome), short-term effects (within seconds to minutes) have also been observed. These rapid non-genomic effects are manifested by the opening of ion channels, the induction of second messengers, the control of phosphatase, kinase and phospholipase activity, etc.

Intensive research into the stereometric properties of the molecule suggests that the spatial arrangement and the location of the VDR are responsible whether vitamin D will trigger genomic or non-genomic actions, respectively [100,101]. In brief, the vitamin D molecule is a seco-steroid with a fractured B-ring, which enables rotation around the carbon 6–7 single bond to obtain a whole range of conformations from 6-s-*cis* (steroid-like) to 6-s-*trans* (extended). As the VDR contains two overlapping ligand-binding sites (a genomic and an alternative binding site), only a molecule with a particular shape fits into the binding site. A bow-like ligand configuration triggers gene transcription, whereas a planar-like ligand shape triggers rapid responses. Further, when measuring the activity of 1,25(OH)2D3 analogs, the 6-s-cis conformation is preferred for rapid non-genomic biological responses, but neither 6-s-cis- nor 6-s-trans-locked analogs are preferred for genomic biological responses [102]. It has been reported that 1α,25(OH)2D3 is able to change its conformation much more quickly than the receptor protein [101]. This selective and specific binding to the VDR represents a model of dynamic regulation that combines genomic and non-genomic signaling by active vitamin D.

### 4.3. Endocrine and Auto-/Paracrine Effects of Vitamin D

The role of vitamin D in maintaining the calcium/phosphate balance and bone health implies a common endocrine model of action involving the renal production of biologically active 1,25(OH)2D3 by 1α-hydroxylase. In addition to the well-accepted endocrine effects of vitamin D, a growing number of studies are suggesting that the local regulation of vitamin D action occurs at the paracrine/autocrine level, as 1α-hydroxylase has been identified in many sites other than renal tissues [18,103]. Therefore, the final activation step in renal tubules represents one of many metabolic pathways to activate vitamin D. The action of vitamin D at the local level is thought to be based on changes in gene expression, the regulation of differentiation, and the cell cycle [18]. Due to the rapid non-genomic effects and paracrine regulation of vitamin D, evaluating the results of clinical studies is complex because they are mostly based on the measurement of circulating total 25(OH)D.

## 5. Conclusions and Future Perspectives

Despite the rapid development of analytical techniques, the measurement of vitamin D is still a major challenge. As the need for examinations grows every year, requirements for the accuracy and speed of tests concurrently increase. Though immunoanalytical methods have the advantage of possible automation, they still produce great variability in inter-laboratory comparisons despite years of effort. Furthermore, methods based on mass spectrometry also suffer from analytical issues, such as a high degree of matrix effects and insufficient analytical sensitivity when measuring less common vitamin D metabolites in physiological conditions, which requires the addition of a derivatization step. Using chromatographic methods, it is possible to distinguish structurally similar derivatives, including epimers. To date, more than 60 different metabolites have been described, but only the biological activity of calcitriol has been fully demonstrated. Vitamin D metabolites constitute a whole network that is comparable to the steroid metabolic network, including precursors, active agents, and catabolites. Similar to steroid hormones, we assume that other forms of vitamin D have biological functions. Indeed, metabolomic studies that evaluate multiple analytes at the same time have proven to be beneficial. Many of these studies have identified previously unknown effects, e.g., the mineralocorticoid activity of deoxycorticosterone [104], or performed metabolomic profiling to facilitate the diagnosis of malignancy [105]. The results on 3-epimers of vitamin D are very promising; these molecules are elevated during pregnancy and presumably do not function as a storage pool because 3-epimerization is an irreversible process. It can be speculated that they may act at much lower concentrations than can be measured by current measurement techniques (recently picograms in milliliter) and act at levels that differ from those involved in the regulation of calcium/phosphate metabolism. With more advanced sensitive assays, it is likely that other vitamin D metabolites will be found in the serum of humans in the future. In vitro studies have indicated that the biological potency of such metabolites is sufficiently high, so circulating concentrations in the lower picogram/milliliter range may be adequate for their significant physiological role.

Importantly, when designing studies, it is advantageous to account for the recently described, non-classical effects of vitamin D. While most current tests detect biologically inactive calcidiol to evaluate vitamin D supply status, active metabolites are not routinely measured. The results of studies may therefore be influenced by metabolic processes that occur between the storage pool and the active form of vitamin D. In addition, it is likely that local auto-/paracrine regulation within vitamin D-responsive microsystems interferes with endocrine mechanisms. It is possible that active metabolites are locally formed from circulating metabolites in the storage pool and locally act within microsystems. If manifested in the circulation, such metabolites could only be determined by highly sensitive detection methods.

The presented paper aims to provide an overview of the main challenges faced in the laboratory. We note that this review does not cover all issues that present difficulties in clinical studies and that may cause many them to fail, such as the unresolved dosing of vitamin D supplementation or insufficient responses to supplementation due to the reduced sensitivity of the VDR.

## Figures and Tables

**Table 1:** Normal ranges of vitamin D metabolites in various biological matrices.

Matrix	Analyte	Method	Average Concentration	Reference
AF	25(OH)D3	LC–MS/MS	0.4–3.2 ng/mL	[59]
	24,25(OH)2D3	RIA	0.017–0.225 ng/mL	[58]
	1,25(OH)2D3	RIA	4.3–24.3 ng/mL	[58]
Breast milk	D3-S	LC–MS/MS	99.28–109.52 pg/mL	[38]
	25(OH)D3	LC–MS/MS	32.04–37.56 pg/mL	[38]
CSF	Total 25(OH)D	RIA	2.0–24.8 ng/mL	[60]
	Total 24,25(OH)2D	RIA	0.3–4.6 ng/mL	[60]
	Total 1,25(OH)2D	RIA	2.2–39 pg/mL	[60]
Serum/plasma	25(OH)D3	LC–MS/MS	30–80 ng/mL	[61]
	25(OH)D2	LC–MS/MS	0.62–5.63 ng/mL	[62]
	3-epi-25(OH)D3 (infant)	LC–MS/MS	0–92 ng/mL	[63]
	3-epi-25(OH)D3 (pediatric)	LC–MS/MS	0–3 ng/mL	[63]
	3-epi-25(OH)D3 (adult)	LC–MS/MS	0–9 ng/mL	[63]
	24,25-(OH)2D3	LC–MS/MS	2–8 ng/mL	[61]
	1α,25(OH)2D3	LC–MS/MS	31–72 pg/mL	[21]
	1β,25(OH)2D3	LC–MS/MS	3–19 pg/mL	[21]
	D2-S	LC–MS/MS	0.192–0.203 ng/mL	[38]
	D3-S	LC–MS/MS	0.267–0.296 ng/mL	[38]
	25(OH)D2-S	LC–MS/MS	0.566–0.613 ng/mL	[38]
	25(OH)D3-S	LC–MS/MS	4.115–4.200 ng/mL	[38]
Synovial fluid	3epi-25(OH)D3	LC–MS/MS	0.832–1.59 ng/mL	[19]
Umbilical cord blood	25(OH)D	RIA	6.1–12.7 ng/mL	[56]
	24,25(OH)2D3	RIA	0.14–1.66 ng/mL	[58]
	1,25(OH)2D3	RIA	10.65–47.78 pg/mL	[58]
Urine	25(OH)D3	LC–MS/MS	3.6–25.2 ng/g creatinine	[41]
	24,25(OH)2D3	LC–MS/MS	11.7–83.2 ng/g creatinine	[41]

Abbreviations: 1,25(OH)2D3: calcitriol; 24,25(OH)2D3: 24,25-dihydroxycholecalciferol; 25(OH)D3: calcidiol; AF: amniotic fluid; CSF: cerebrospinal fluid; D2-S: ergocalciferol sulfate; D3-S: cholecalciferol sulfate; LC–MS/MS: liquid chromatography–tandem mass spectrometry; RIA: radioimmunoassay.

**Table 2:** Variations in vitamin D extraction.

Matrix (Amount/Assay)	PP	Extraction	Method	Stationary Phase	Reference
Plasma and urine	MeCN	SPE Sep-Pak C18 cartridges		NP Silica	[64] *
Plasma (0.5 mL)	EtOH	n-hexane/CH2Cl2 (90:10, *v*/*v*)	HPLC–UV/VIS	C18	[65]
Serum (0.5 mL)	EtOH isopropanol/MeOH (1:9, *v*/*v*)	n-hexane	HPLC–UV/VIS	C18	[66]
Animal/human plasma (1.5 mL)	EtOH + MeOH KOH +ASC	heptane	HPLC–UV/VIS	C30	[67]
Serum (0.25 mL)	EtOH	n-hexane Sephadex LH-20 fractionation	ID-LC–MS/MS	C4 and C18 (CN resp.)	[68]
Serum (ca. 2 g)	EtOH	hexane/EA (50:50, *v*/*v*)	ID-LC–MS/MS	CN	[69]
Plasma (0.2 mL)	EtOH	n-hexane	HPLC–UV/VIS	C18 and C30	[70]
Serum (0.1 mL)	EtOH	MTBE	LC–MS/MS	F5	[71]
Serum (50 µL)		SLE–TICE (AC Extraction Plate)	LC–MS/MS	F5	[72]
Dried blood spot (ca. 6 µL)	MeOH		LC–MS/MS (deriv. by DAPTAD)	F5	[73]
Dried blood spot (ca. 3 µL)	MeCN	EA/CH2Cl2 (1/3, *v*/*v*)	LC–MS/MS (deriv. by INC)	F5	[57]

Abbreviations: ASC: acid-solubilized collagen; C4, C18, and C30: number of carbon atoms of the stationary phase; CH2Cl2: dichloromethane; CN: stationary phase bonded to cyanopropyl functional groups; DAPTAD: 4-(4′-dimethylaminophenyl)-1,2,4-triazoline-3,5-dione; EA: ethyl acetate; EtOH: ethanol; F5: pentafluorophenyl propyl phase; HPLC–UV/VIS: high-performance liquid chromatography with ultraviolet or visible light detection; ID: isotope dilution; INC: isonicotinoyl chloride; LC–MS/MS: liquid chromatography–tandem mass spectrometry; KOH: potassium hydroxide; MeCN: acetonitrile; MeOH: methanol; MTBE: methyl tert-butyl ether; NP: normal phase; PP: protein precipitation; SLE: supported liquid extraction; SPE: solid-phase extraction; TICE: Tecan Immobilized Coating Extraction. * poor recovery reported.

**Table 3:** Recommended vitamin D optimal levels based on various sources.

Author	Serum Total 25-Hydroxyvitamin D (ng/mL)	Subjects	Reference
Institute of Medicine	20–50 ng/mL	Adults	[97]
Endocrine Society	30–100 ng/mL	Adults	[98]
Hollis and Wagner	>40 ng/mL *	Pregnant women	[93]
Dawson-Hughes et al.	>40 ng/mL *	Elderly	[99]

* Maximum level not reported.

## Data Availability

Excluded as the review does not report any measured data.
